# Clinically feasible brain morphometric similarity network construction approaches with restricted magnetic resonance imaging acquisitions

**DOI:** 10.1162/netn_a_00123

**Published:** 2020-03-01

**Authors:** Daniel J. King, Amanda G. Wood

**Affiliations:** School of Life and Health Sciences and Aston Neuroscience Institute, Aston University, Birmingham, United Kingdom; School of Life and Health Sciences and Aston Neuroscience Institute, Aston University, Birmingham, United Kingdom; School of Psychology, Faculty of Health, Melbourne Burwood Campus, Deakin University, Geelong, Victoria, Australia

**Keywords:** Morphometric similarity networks, Structural MRI, Morphology, Connectome, Cognition

## Abstract

Morphometric similarity networks (MSNs) estimate organization of the cortex as a biologically meaningful set of similarities between anatomical features at the macro- and microstructural level, derived from multiple structural MRI (sMRI) sequences. These networks are clinically relevant, predicting 40% variance in IQ. However, the sequences required (T1w, T2w, DWI) to produce these networks are longer acquisitions, less feasible in some populations. Thus, estimating MSNs using features from T1w sMRI is attractive to clinical and developmental neuroscience. We studied whether reduced-feature approaches approximate the original MSN model as a potential tool to investigate brain structure. In a large, homogenous dataset of healthy young adults (from the Human Connectome Project, HCP), we extended previous investigations of reduced-feature MSNs by comparing not only T1w-derived networks, but also additional MSNs generated with fewer MR sequences, to their full acquisition counterparts. We produce MSNs that are highly similar at the edge level to those generated with multimodal imaging; however, the nodal topology of the networks differed. These networks had limited predictive validity of generalized cognitive ability. Overall, when multimodal imaging is not available or appropriate, T1w-restricted MSN construction is feasible, provides an appropriate estimate of the MSN, and could be a useful approach to examine outcomes in future studies.

## Author Summary

We can estimate the higher order organization of cortical gray matter as a connectome using structural MRI. However, this methodology, termed morphometric similarity, requires multiple advanced neuroimaging protocols that are unsuitable, unavailable, or intolerable to certain populations, including children and some clinical groups. In a large, homogenous dataset of healthy young adults, we estimated these connectomes using three different feature sets, each extracted from fewer MRI sequences. Even when produced using only T1-weighted structural MRI scans, these connectomes were broadly similar to those produced with more complex or numerous MRI sequences. We did not replicate previous findings linking variation in the morphometric similarity networks (MSNs) to individual differences in cognitive abilities. We highlight potential reasons for this, including the developmental stage of the young adult imaging cohort in which our hypotheses were tested, and conclude that this study provides putative evidence that, in those populations where advanced imaging is not plausible, MSNs generated from T1-weighted structural MRIs are a promising alternative.

## INTRODUCTION

Cortical gray matter structural covariance networks (SCNs) model the degree to which the morphology of cortical regions (e.g., cortical thickness or volume) statistically covaries across all possible pairs of regions (Alexander-Bloch, Giedd, & Bullmore, 2013; Evans, 2013). These whole-brain network approaches to morphometry, within a graph theoretic framework, allow us to investigate additional information beyond univariate approaches to neuroanatomy (Pagani, Bifone, & Gozzi, 2016). Disruption to the SCN has been explored in a range of neurological and neuropsychiatric conditions. However, this methodology necessarily generates group-level networks indexing population-level covariance in neuroanatomy (Alexander-Bloch, Raznahan, Bullmore, & Giedd, 2013). This limits the ability to quantify system-level abnormalities within individual patients, who could benefit from stratified diagnosis and prognosis (Zheng, Yao, Xie, Fan, & Hu, 2018).

An alternative approach to investigate the regional covariance structure between multiple morphometric features at an individual level is morphometric similarity networks (MSNs; Seidlitz et al., 2018). This approach estimates mesoscale organization of the cortex as a biologically meaningful set of similarities between anatomical features at both the macro- and the microstructural level (Morgan et al., 2018), including mesoscale morphometry, tissue diffusion properties, and myelination indices. MSNs have been shown to be clinically useful, predicting ∼40% variance in IQ, as well as biologically meaningful, with edges of the MSN highly aligned with regional gene coexpression in human data and axonal tract tracing in the rhesus macaque (Seidlitz et al., 2018). These findings reflect the fact that cortical regions that are more anatomically similar are likely to be anatomically connected (Goulas, Uylings, & Hilgetag, 2017; Wei, Scholtens, Turk, & van den Heuvel, 2019). The MSN represents a new neuroimaging phenotype, which may provide additional, biologically relevant information beyond existing network approaches.

MSNs have already been utilized in a small number of clinical studies (characteristics of these studies, including neuroanatomical features extracted, are listed below in Table 1). For example, previous studies identified a robust, replicable pattern of differences in morphometric similarity between patients with psychosis compared with controls (Morgan et al., 2018) and detected dysmaturation of the brain in preterm infants (Galdi et al., 2018). Overall, these findings suggest that MSNs represent a useful and clinically relevant phenotype.

**Table 1.** Characteristics of previous studies utilizing morphometric similarity networks to investigate cortical organization

Study	Population	MRI acquisitions	MSN feature set
Seidlitz et al. (2018)	Typically developing adolescents	Multiparametric mapping sequence and DWI	FA, MD, MT, GM, SA, CT, GC, MC, CI, FI (Reduced-feature MSN; CT, SA, GM, GC, MC)
Morgan et al. (2018)	Schizophrenia or nonaffective psychotic disorders	T1w MRI and DWI	GM, SA, CT, GC, MC, FA, MD
Galdi et al. (2018)	Term and preterm neonates	T1w MRI, T2w MRI, and DWI	GM, T1/T2 ratio, FA, MD, RD, AD, VISO, IVF, ODI
Seidlitz et al. (2019)	Neurodevelopmental disorders of known genetic origin	T1w MRI	CT, SA, GM, MC, GC
Li et al. (2017)	Healthy adults	T1w MRI	Vertices, GM, SA, CT, SD-CT, MC, GC, CI, FI
Zheng et al. (2018)	Mild cognitive impairment and Alzheimer’s disease	T1w MRI	CT, SA, GM, LGI, sulcul depth, gyrus height
Zheng et al. (2019)	Autism spectrum disorders	T1w MRI	CT, SA, GM, LGI, sulcul depth, gyrus height, MC

*Note*. FA = fractional anisotropy, MD = mean diffusivity, MT = magnetization transfer, GM = gray matter volume, SA = surface area, CT = cortical thickness, GC = Gaussian curvature, MC = mean curvature, CI = curvature index, FI = folding index, RD = radial diffusivity, AD = axial diffusivity, VISO = isotropic volume fraction, IVF = intracellular volume fraction, ODI = orientation dispersion index, vertices = number of vertices, SD-CT = standard deviation of cortical thickness, LGI = local gyrification index.

Multimodal, high-quality MRI sequences are required for these approaches. These may not be feasible or tolerable for all research settings and/or populations. Existing (legacy) clinical cohorts often lack advanced imaging. Longer acquisition times for advanced sequences may reduce image quality, especially in pediatric or clinical applications where noncompliance and movement are more likely as acquisition time increases (Rosen et al., 2018).

Estimating MSNs using a single T1w 3-D anatomical MRI is appealing for clinical and developmental neuroscience (Batalle, Edwards, & O’Muircheartaigh, 2018). Seidlitz et al. (2018) and Li et al. (2017) estimated morphometric similarity in this way and found that network edge weights were similar to multimodal MSNs (r = 0.68; Seidlitz et al., 2018), with good test-retest reliability in terms of network topology (ICC = 0.60; Li et al., 2017). Using only T1w MRI it is possible to identify patterns of morphometric similarity that classify autism spectrum disorder (Zheng et al., 2019), Alzheimer’s disease, and mild cognitive impairment (Zheng et al., 2018) from controls, albeit with reduced estimation precision, and greater standard deviation of edge-level weights across participants (Seidlitz et al., 2018). This method also identified a spatial pattern of anatomical disruptions associated with regional gene expression, findings consistent with a transcriptional vulnerability model of neurodevelopmental disorders (Seidlitz et al., 2019). Characteristics of these studies, including neuroanatomical features extracted, are listed below in Table 1. However, existing studies provide limited assessment of reliability, consistency with group networks, biological validity, and predictive ability.

The current study aimed to determine whether reduced-feature approaches approximate the original MSN model. We extended previous investigations of reduced-feature MSNs by comparing not only T1w-derived networks, but also additional MSN models, each using fewer metrics from a reduced number of MRI acquisitions.

We predicted that for each measure of reliability/replicability, performance would be ordered hierarchically with MSNs generated with the greatest number of features outperforming those generated with fewer. We also predicted that between-model comparisons would suggest that the models themselves were highly similar. In line with previous work (Seidlitz et al., 2018), we predicted that there would be an association between cognition and MSN organization and that this would generalize to a novel cognitive domain, specifically executive functioning.

## METHODS

### Participants: HCP Data

The current study uses open-access, 3T MRI data provided by the Human Connectome Project (Van Essen et al., 2013), shared via ConnectomeDB (https://db.humanconnectome.org) under the HCP 1200 and HCP test-retest release. Favorable ethical approval for the secondary analysis of this data was granted by the Aston University ethics panel.

#### HCP 1200 release.

The HCP 1200 release contains data from *n* = 1,206 subjects (550 males, 656 females). Subjects are grouped into age bins from 22–25 to 36+ (median age = 26–30). While *n* = 1,206 subjects provided behavioral data, only 1,113 subjects had MRI data available. These were the subjects for which data were accessed and downloaded from ConnectomeDB for the current study.

#### HCP test-retest release.

For 46 subjects from the HCP 1200 release, a second retest dataset is available to assess test-retest reliability of analyses. These second MRI visits occurred within time bins from 1–2 months to 11 months post initial scanning session. The median retest-interval bin was 5 months. Of these subjects, 45 had available MRI data, and these were the subjects used for subsequent analyses.

### Methods

#### Data quality control.

Subjects were selected for inclusion if, in the 1,200-subject HCP release, they had T1w (0.7 mm isotropic), T2w (0.7 mm isotropic), and diffusion data uploaded. This led to exclusion of *n* = 76 cases.

Also, utilizing QC data shared by the Human Connectome Project, any data labeled with QC issue code B (which flags cases as having focal segmentation and surface errors when the corresponding FreeSurfer outputs were checked) were further excluded from the current study (*n* = 33). The final dataset consisted of *n* = 1,004 subjects. In the test-retest cohort, only one subject was excluded as flagged with QC issue code B by the Human Connectome Project.

#### MRI processing.

The current study utilizes data shared in preprocessed format, including the output of the HCP FreeSurfer pipeline (Fischl et al., 2002; Glasser et al., 2013; Jenkinson, Bannister, Brady, & Smith, 2002; Jenkinson, Beckmann, Behrens, Woolrich, & Smith, 2012), processed DWI (gradient nonlinearity, eddy current, and EPI distortion corrected; Andersson, Skare, & Ashburner, 2003; Andersson & Sotiropoulos, 2015, 2016), and calculated T1/T2w ratio myelin maps (Glasser & Van Essen, 2011). For further details of HCP processing pipelines see Glasser et al. (2013).

Once cases were selected, measures indexing the underlying neuroanatomical structure were derived from multiple imaging modalities (see Table 2). Seidlitz et al. (2018) leverage near-identical MRI-derived metrics for the construction of the MSN network. However, we are using the T1/T2 ratio as a proxy for myelin content, rather than the magnetization transfer scan used by Seidlitz et al. (2018). The rationale for this modification was both pragmatic and clinically driven; (a) the T1/T2w ratio maps are already implemented by the Human Connectome Project and thus these data are available for use with the rest of the high-quality HCP acquisition data; and (b) in clinical populations, for which the methods may provide greatest benefit, multiparameter mapping MRI sequences may not be acquired as part of a clinical protocol, whereas T1w and T2w sequences are.

**Table 2.** Morphometric measures and the modality of MRI from which they were derived

Modality	Metrics
T1w	Cortical thickness (CT), surface area (SA), mean (extrinsic) curvature (MC), Gaussian (intrinsic) curvature (GC), folding index (FI), curvature index (CI), and gray matter volume (GM)
T2w	Myelination (T1/T2w ratio)
DWI	Fractional anisotropy (FA), mean diffusivity (MD)

Preprocessed DWI (1.25 mm isotropic, *b* = 1,000) in T1w space were fitted to a tensor model using FMRIB’s “dtifit” function, and the subsequent FA and MD maps were mapped to the individual subject’s FreeSurfer-generated surface model in MNI space, using the connectome workbench (Marcus et al., 2011) function “volume-to-surface-mapping.” These, and the Tw1/T2w ratio myelin maps, were parcellated based on the Desikan-Killiany atlas (Desikan et al., 2006), by generating a dense-cifti (using the “cifti-create-dense-from-template” function) and parcellating the output (using “cifti-parcellate”). FreeSurfer metrics were also extracted for each parcellated region using the “aparcstats2table” function.

#### MSN construction.

To generate MSNs we apply the methods of Seidlitz et al. (2018) to the HCP data. The Desikan-Killiany atlas was mapped to the individual subjects with a surface-based registration, using the FreeSurfer pipeline. The Desikan-Killiany atlas regions of interest (ROIs) were used as the nodes for all network construction.

Morphometric features (parcellated to the Desikan-Killiany atlas) for each participant can be expressed as a set of n vectors of length 10, with each vector as a different anatomical region (*n* = 68), and each element of the vector a different morphometric measure. However, these features are not all measured at the same magnitude of scale. For instance, volume (mm^3^) is measured at the order of 10^3^, whereas folding index is measured to the order of 10^1^. Thus, to normalize within this length 10 vector, each of these morphometric features is normalized across the 68 regions, using Z-scores (demeaned and SD scaled). This brings the measures across the feature vector into a comparable range.

Using the normalized features, a correlation matrix is generated for each participant, where each element of the matrix is the correlation between the feature vectors for every possible pairwise combinations of regions. Because each feature is zero-centered, the resultant distribution of correlation coefficients is normally distributed about zero. This correlation matrix represents the MSN-estimated connectivity for each participant.

We constructed these networks across three different MSN models. These models were hierarchically organized, with reduced acquisition complexity from Models A to C below:

(A)  MSN (T1w + T1w/T2w ratio + DWI; 10 features (MSN_10-feat._)),
(B)  MSN (T1w + T1w/T2w ratio; eight features (MSN_8-feat._)),
(C)  MSN (T1w; seven features (MSN_7-feat._))

Model A, hereto referred to as MSN_10-feat._, is the best approximation of the Seidlitz et al. (2018) approach, with magnetization transfer replaced with T1w/T2w ratio mapping (Glasser & Van Essen, 2011) in the current study. Thus, for each participant, three MSNs (one per model) were estimated.

### Demographic and Behavioral Data

Demographic variables were selected from the unrestricted data table accessed via ConnectomeDB. These included age bin, sex recorded at birth, and recorded quality control issues. Behavioral data were also extracted to assess the relationship between the MSNs and both general cognitive ability (measured with both fluid and crystallized intelligence measures) and executive functioning. These neuropsychological assessments were conducted contemporaneously in relation to the MRI scans. Further details of the tasks and measures acquired in the HCP dataset can be found in Barch et al. (2013).

#### General cognitive ability.

General cognitive functioning is measured with the Cognitive Function Composite (CogComp) score (Heaton et al., 2014), derived from the average of the normalized, scaled scores of Fluid and Crystallized cognition measures, then subsequently age adjusted, and scaled. The Fluid Cognition Composite score is derived by averaging the normalized scores of each of the fluid ability measures in the NIH Toolbox (Flanker, Dimensional Change Card Sort, Picture Sequence Memory, List Sorting, and Pattern Comparison), while the Crystallized Cognition Composite score is derived by averaging the normalized scores of each of the crystallized measures in the NIH Toolbox (Picture Vocabulary and Reading Tests). Higher Cognitive Function Composite scores indicate higher levels of cognitive functioning.

#### Executive functioning.

Behavioral executive function (EF) measures were selected based on an evidence-based, three-factor model of executive function (Karr et al., 2018); measures selected from the HCP cognitive battery to model EF were the same as previous studies of EF utilizing the HCP data (Lerman-Sinkoff et al., 2017; Nomi et al., 2017). These tests assessed multiple cognitive aspects of executive functioning, including cognitive flexibility/shifting (Dimensional Change Card Sort Test; Zelazo, 2006; Zelazo et al., 2014), inhibition (Flanker Inhibitory Control and Attention Task; Zelazo et al., 2014), and working memory (List Sorting Task; Tulsky et al., 2013). Age-adjusted scores were used for all behavioral data.

Since we have only one neuropsychological measure per subdomain of EF and there is therefore potential risk of measurement error, a principal component analysis (using the “prcomp” function in the R stats base package; R Core Team, 2016) was used to find a common EF component across all three EF measures. This produced a single principal component with an eigenvalue above 1, upon which all measures positively loaded onto, and thus this component was used as a summary score of EF (see the Supporting Information for further details). Higher summary EF scores reflect greater EF functioning.

### Statistical Comparison

When comparing weighted networks produced by each model, we use multiple metrics to assess the (dis)similarity of the subsequent covariance matrices.

To reduce the number of comparisons and, based on our premise that the MSN_10-feat._ is the most precise estimation of the MSN network (as shown by Seidlitz et al., 2018), all intermodel comparisons were done in a hierarchical fashion in comparison to this “gold-standard” network. That is to say that model MSN_10-feat._ was compared with the MSN_8-feat._ and then the MSN_10-feat._ was subsequently compared with the MSN_7-feat._.

To test differences in the topological organization of the networks produced by each model, we calculate average nodal strength for each graph. Nodal strength is the magnitude of structural covariance for each node; that is, the sum of the connectivity weights of all edges connected to node i (Fornito, Zalesky, & Bullmore, 2016). We did not normalize this measure based on number of edges as we averaged the nodal measures over the graph, where the number of edges was consistent across models because of density thresholding. This metric was calculated per subject, per density for each MSN model. For each comparison, we calculate the difference in distributions of graph strength using a paired t test. Because of the large number of comparisons (across densities, and contrasts) we do not report p values, but instead report the effect sizes for comparisons.

We also calculate the Pearson correlation coefficient between all edge weights for both models (as per Seidlitz et al., 2018), and also specifically between all nonzero edge weights (those elements where a zero is present in the correlation matrix for each model are excluded). However, because of the symmetric, undirected nature of the correlation matrix, this correlation coefficient may inflate/bias the supposed similarity between the sets of edge weights. Thus we also employed the Mantel test, which calculates the Pearson correlation on either half of the off-diagonal elements of the correlation matrix (Mantel, 1967).

To compare the binary networks produced by each model at each density (where edges retained after thresholding are set to 1 and those excluded are set to 0), we assessed the number of edges in the reduced model that replicated as a proportion of the fuller model, as per the following formula:

$$\frac{\sum (x_{i}\neq 0\;\&\;y_{i}\neq 0)}{\sum (x_{i}\neq 0)},$$

where *x*_*i*_ and *y*_*i*_ represent the correlation matrices estimated from two of the MSN models for a given subject *i*.

Second, we calculate these similarity measures between the subject-level network and the group average network, across all densities and models. This allows the assessment of the intersubject reliability of the networks being constructed by each model. Third, we similarly test the intrasubject reliability of the produced networks, based on test-retest data from a subset of the overall dataset. Because of the categorical and inaccurate nature of the binned measurement of time between initial and retest scan, this was not controlled for in this analysis.

In order to assess the functional relevance of these networks, we assess their ability to predict CogComp and EF scores using a supervised-learning approach, namely partial least squares (PLS) regression (similar to Seidlitz et al., 2018) using the “plsRglm” package in R (Bertrand & Maumy-Bertrand, 2018). This multivariate approach finds the optimal low-dimensional relationship between a high-dimensional set of predictors (in this case the MSN networks) and a univariate predictor variable (either CogComp or EF). This approach is commonly used when the number of predictors exceeds the number of observations (Krishnan, Williams, McIntosh, & Abdi, 2011).

A PLS regression was used to find the maximal low-dimensional covariance between components derived from the MSN and cognitive outcomes. The PLS regression was used to decompose the predictor variables into latent variables (components) that simultaneously model the predictors and predict the response variable (Krishnan et al., 2011). The predictor matrix consisted of either the degree or the strength of each node of the MSN, for each participant. Using a linear model, the potential confounding effect of age, gender, and age × gender interaction was regressed out of values for nodal degree/strength (but not our cognitive outcome variable as these were already age-adjusted within the HCP dataset). For each model (at each threshold), a PLS regression model was fitted between principal components derived from the resultant predictor matrix (68 × 991) and the outcome variable. This was repeated across 100 instances of ninefold cross-validation.

Cross-validated *R*^2^ (*R*^2^_*CV*_), otherwise known as the *Q*^2^ statistic (Consonni, Ballabio, & Todeschini, 2010; Stone, 1974), was used to select the number of components to retain in the predictor matrix. *Q*^2^ was defined as the following:

$$Q^{2}={R^{2}}_{CV}=1-\frac{PRESS}{TSS}=1-\frac{\sum_{i=1}^{n}{\left({ŷ}_{i}-y_{i}\right)}^{2}}{\sum_{i=1}^{n}{\left(y_{i}-\overset{-}{y}\right)}^{2}},$$

where *PRESS* is the predictive residual error sum of squares and *TSS* is the total sum of squares.

The number of components to retain in the predictive model was selected as the number of components that resulted in the greatest *Q*^2^ value. This was repeated over the cross-validations and resulted in a count measure of the number of times a model with a given number of components was selected. Hence the final model was the given number of components that were most commonly selected as having the greatest *Q*^2^ statistic. Given the model with the retained number of components, we report the variance explained by the model and the bias corrected and accelerated bootstrapped (Bastien, Vinzi, & Tenenhaus, 2005) weightings of each predictor. This allows us to assess which brain regions are contributing most to the prediction.

Because of the normal distribution of the cognitive measures (CogComp and EF) data, there may be an issue of class imbalance for more extreme cases (Torgo, Branco, Ribeiro, & Pfahringer, 2015). As there are fewer subjects who fall within the tails of the continuous distribution on our cognition measures, the cross-validation approach may lead to training samples where there are too few extreme cases (those with particularly high/low cognitive abilities) to learn from. This may result in a model where there is accurate prediction around the mean but not at the tail ends of the distribution. To ensure the training samples contain subjects from a stratified sampling approach, we repeated the analyses discretizing the performance on cognitive measures into four discrete bins across the distribution and training a model based on equally sized, random samples from each bin.

## RESULTS

### Intermodel Comparisons

#### Magnitude of morphometric similarity: Graph-level strength.

**Figure 1.**
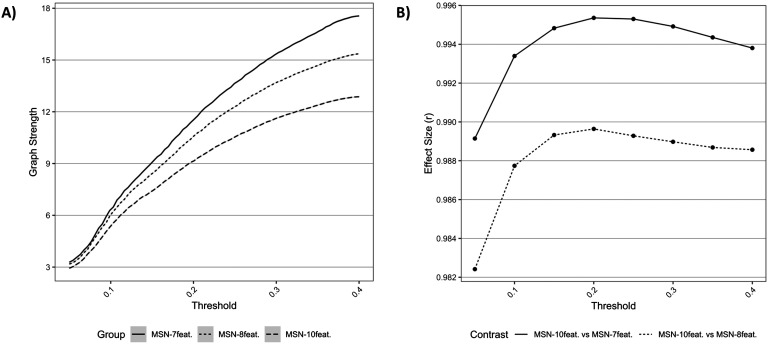
Comparisons of network topology. (A) Graph metrics describing average network strength for each MSN model, across all densities. (B) Effect sizes of differences between (a) MSN_10-feat._ versus MSN_8-feat._ and (b) MSN_10-feat._ versus MSN_7-feat._ for differing graph metrics, across densities.

In terms of the topology of the networks, global graph strength for each model, across densities, can be seen in Figure 1. This plot shows the similar trajectories across densities for all models tested; however, the observed average graph strength was different between models, with lower strength seen in the MSN models with greater features. The effect size of differences (estimated with a paired *t* test) between MSN_10-feat._ versus MSN_8-feat._ and MSN_10-feat._ versus MSN_7-feat._ can be also be seen in Figure 1. Effect sizes (*r*) were extremely large, especially between MSN_10-feat._ versus MSN_7-feat_.

#### Edge weights.

**Figure 2.**
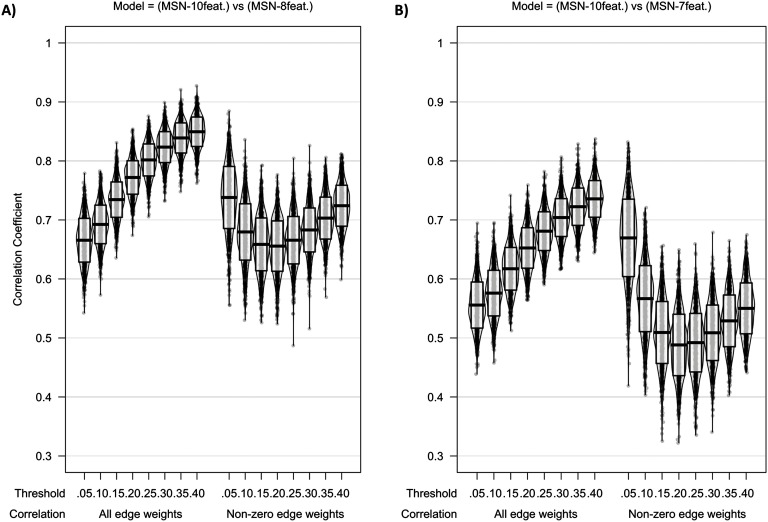
Violin plot of correlation of edge weights between (A) MSN_10-feat._ versus MSN_8-feat._ and (B) MSN_10-feat._ versus MSN_7-feat._ Midline of the box plot component of the violin represents the mean of all correlation coefficients, with the box itself representing the SD of this mean. Individual data points are also plotted.

Figure 2 shows the intermodel comparisons between MSN_10-feat._ and MSN_8-feat._, and between MSN_10-feat._ and MSN_7-feat._. There is a gradual increase in correlation of edge weights across densities with the peak mean correlation being found between MSN_10-feat._ and MSN_8-feat._ at a 40% threshold (*r*(*M* ± *SD*) = 0.849(± 0.025)), with slightly weaker correlations found between MSN_10-feat._ and MSN_7-feat._ (*r*(*M* ± *SD*) = 0.736(± 0.031)). When considering only the nonzero edge weights (only edge weights remaining after thresholding), a slightly weaker peak correlation was found for both contrasts at 5% threshold (MSN_10-feat._ versus MSN_8-feat._ *r*(*M* ± *SD*) = 0.738(± 0.053); MSN_10-feat._ versus MSN_7-feat._ *r*(*M* ± *SD*) = 0.670(± 0.066)). However, as the threshold increased, the dispersion of individual-level nonzero edge correlation decreases, especially in the MSN_10-feat._ versus MSN_7-feat._ contrast.

**Figure 3.**
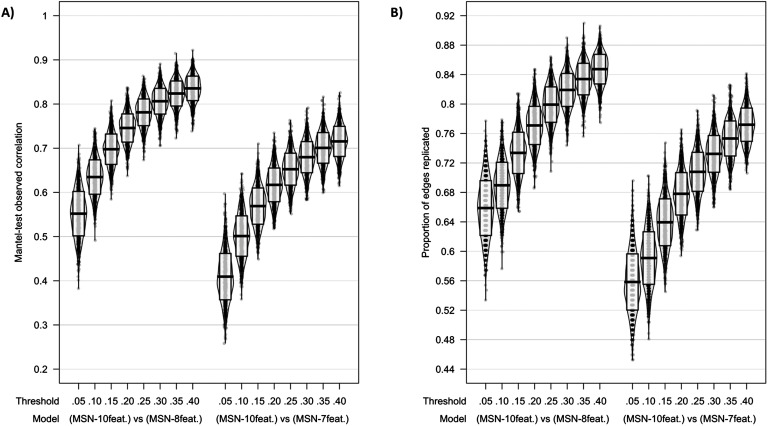
Model comparisons across thresholds using (A) Mantel test correlation coefficient and (B) proportion of edges replicated as measures of model similarities. Midline of the box plot component of the violin represents the mean, while the box itself represents the SD.

When considering correlation coefficients calculated using the Mantel test, similarly strong correlations were found between edge weights across all models; however, as predicted, the MSN_10-feat._ versus MSN_8-feat._ were most similar. At 40% threshold: MSN_10-feat._ versus MSN_8-feat._ Mantel *r*(*M* ± *SD*) = 0.835(± 0.028); MSN_10-feat._ versus MSN_7-feat._ Mantel *r*(*M* ± *SD*) = 0.715(± 0.034). For the binarized networks, the proportion of edges replicated also peaked at 40% threshold (MSN_10-feat._ versus MSN_8-feat._ proportion of replicated edges = 85%(± 2%); MSN_10-feat._ versus MSN_7-feat._ proportion of replicated edges = 77%(± 2%; Figure 3)).

### Intramodel Comparisons

#### Test-retest reliability of MSN models.

We compared the MSN models at the initial scan with those calculated from test-retest scans acquired between 1 and 11 months after the initial MRI. All models showed high test-retest reliability of the MSN (correlation of all edge weights at 40% threshold: MSN_10-feat._ *r*(*M* ± *SD*) = 0.902(± .032); MSN_8-feat._ *r*(*M* ± *SD*) = 0.881(± 0.040); MSN_7-feat._ *r*(*M* ± *SD*) = 0.857(± .043)). This high test-retest reliability of networks held even when networks were binarized. At 40% threshold: MSN_10-feat._ proportion of replicated edges = 87%(± 3%); MSN_8-feat._ proportion of replicated edges = 87%(± 3%); MSN_7-feat._ proportion of replicated edges = 86%(± 3%). See Figure 3 for plots.

#### Similarity with average MSN.

**Figure 4.**
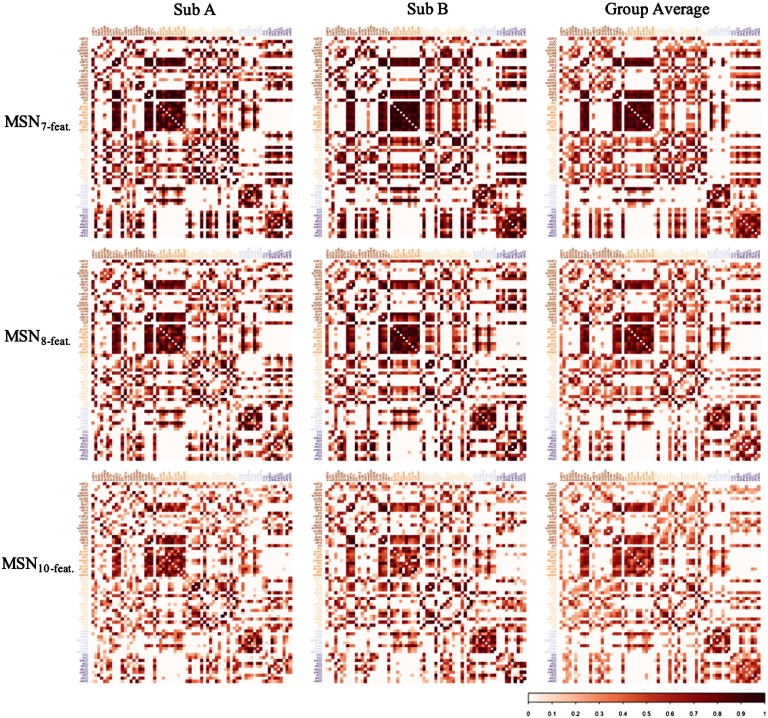
Examples of the correlation matrices generated with each MSN model. Columns A and B represent MSNs from two random subjects from the HCP dataset, while the final column represents the group average MSN for each MSN model. All correlation matrices visualized here represent the MSN thresholded at a density of 40% and are sorted by lobe assignment as defined by the Desikan-Killiany atlas (frontal, parietal, temporal, occipital, insula, cingulate).

**Figure 5.**
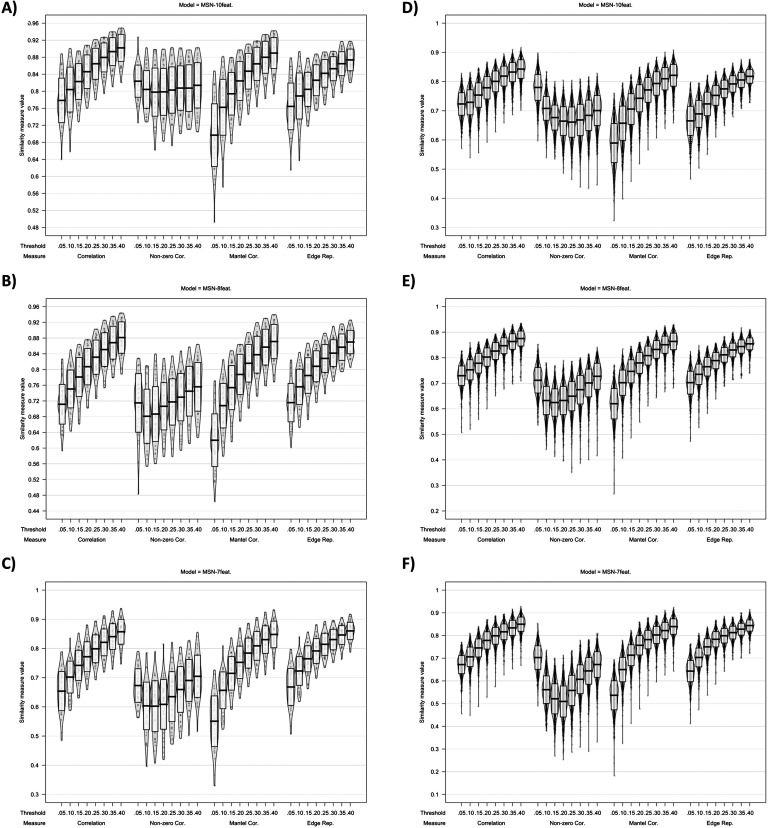
Plots showing MSN similarity (across thresholds, with multiple similarity measures) between (A–C) individual MSNs generated with test-retest MRI scans and (D–F) individual-level MSNs and the group average MSN network.

For each model, at each threshold, a group-level network was produced as the mean of the correlation matrices for all subjects. Example correlation matrices are found in Figure 4. Across all models (MSN_10-feat._, MSN_8-feat._, and MSN_7-feat._), regardless of similarity metric used, the individual-level MSNs were highly similar to the group-mean network (see Figure 5). Interestingly, the MSN_8-feat._ model showed greatest correlation between edge weights. At 40% threshold: MSN_10-feat._ *r*(*M* ± *SD*) = 0.843(± 0.032); MSN_8-feat._ *r*(*M* ± *SD*) = 0.875(± 0.029); MSN_7-feat._ *r*(*M* ± *SD*) = 0.850,(± 0.031). Similar to the intermodel analyses, correlation peaked at the highest threshold tested (40%) for all models.

### Relationship With Cognitive Scores

Only participants who had available a full dataset comprising the three EF subtests and the CogComp measure were included in the following analyses (*n* = 991). For both cognitive variables, using 100 instances of ninefold cross-validation, the greatest *Q*^2^ was found most frequently when zero components were retained and thus no models were built.

This suggests that no PLS-derived components of nodal degree, strength, or normalized strength of the MSN provided greater explanation than the intercept alone. After the stratified sampling of the training cohort, there was no improvement in the result outlined above; cross-validation still recommended retention of zero components for all MSN models.

## DISCUSSION

The current study is the first to formally investigate the potential for generation of MSNs based upon a reduced number of neuroanatomical features, dependent on the complexity of the MRI acquisition sequence. We found that the weighted networks generated from these models are highly congruent, across a number of similarity measures. We expanded previous assessments (e.g., Seidlitz et al., 2018, compared five morphometric features to their full MSN_10-feat._model) to include multiple MSN models. Irrespective of the statistical assessments used here, the between-model similarity was nearly always hierarchical, with greater similarity seen between MSN_10-feat._ and MSN_8-feat._ compared with that between MSN_10-feat._ and MSN_7-feat._ Weaker similarity was found for sparser networks at a much lower density (i.e., 0.05). When edges were binarized, replication rates remained relatively high, suggesting that the models are sensitive to the specific edges within the network. Each model displayed high levels of congruence with the group average network, suggesting that these methods index individual differences from a relatively consistent mesoscale phenotype of brain structure. Li et al. (2017) found high levels of test-retest reliability of the T1w MSN, and we replicated this with each of the reduced-feature MSNs, which seemingly had similar reproducibility in terms of test-retest MRI. This is maybe unsurprising given that the HCP scan acquisitions are designed for high within-subject reproducibility (Van Essen et al., 2013). However, in terms of average nodal strength, a measure of the magnitude of morphometric similarity, significant between-model differences with large effect sizes were found. Specifically, as myelo-architectural features were added to the MSN model (T1w/T2w ratio, FA, MD), the magnitude of morphometric similarity was reduced and regions appeared less similar and more differentiated, hence the lower average nodal strength. This suggests that, despite edge-level congruence, the weighted topology of individual nodes is different between models. Given that the current and previous studies (i.e., Seidlitz et al., 2018) utilize these nodal-level metrics to predict functional outcomes, this difference in topology means that between-study comparisons of these predictive models, when generated from different feature sets, is not justified. These differences were investigated with only a single metric of network topology. Additional metrics (i.e., efficiency/clustering) were less attractive candidates given that the MSNs do not adhere to typical assumptions of networks (such as edges representing definitive real connections). Hence, by using strength as a simpler metric we made fewer assumptions about the underlying neurophysiology of the network.

Interestingly, none of the models tested here showed perfect or even near-perfect concordance across the statistical measures employed. This may be because these models were generated with fewer features, rather than being specific to the modality of feature being dropped. Future research could examine this systematically by generating MSNs with 10, 8, and 7 randomly selected features, irrespective of modality of MRI sequence used to derive said feature.

Each model may also index a different network phenotype. Each MRI acquisition assesses different neuroanatomical features (e.g., T1w/T2w ratio and DWI specifically index myelo-architecture of regions while the T1w MRI assesses macroscopic morphology of the cortex). Thus, when features are systematically removed in this way by removing an imaging modality, the resultant MSN is likely to represent a different imaging phenotype. Therefore, although each MSN may be substantially statistically similar, in cases where multimodal imaging is available or is feasible, the most appropriate MSN model may depend on the research question to be answered or the clinical population/pathological mechanism under investigation.

The main benefit of reduced MR-acquisition approaches (specifically the MSN_7-feat._ model) is the applicability to those populations where multiple MR sequence acquisition is more challenging or difficult (Batalle et al., 2018). For instance, in clinical populations where research MRI scans are acquired alongside routine examinations and therefore time is limited, or in developmental populations where acquisition time needs to be kept at a tolerable level for children to ensure compliance with the full MRI protocol and reduce the likelihood of movement across each of the scans. Another potential application of these models is in legacy clinical imaging. Routine clinical imaging generates vast quantities of MR data that are not typically assessed using quantitative methods. Although expert reporting yields the information needed to inform acute diagnostic requirements, the ability of those scans to predict later outcomes is largely untested or unsatisfactory. The majority of hospital settings will not have access to high-resolution, so-called advanced, MRI sequences, or the expertise to analyze such data quantitatively. Overall, this therefore positions MSNs as a useful in vivo imaging phenotype for studying both clinical and developmental populations, with the T1w-only model holding greatest potential to become a widely adopted, automated approach in clinical neurosciences.

A common assumption is that the topological organization of the brain networks (across multiple MR modalities), as quantified within a graph theoretic framework, captures physiological information relevant to individual differences in cognitive functioning (Bullmore & Sporns, 2009; Fornito, Zalesky, & Breakspear, 2013; Hahn, Lanzenberger, & Kasper, 2019). We assessed the brain-behavior relationships using the MSN models by comparing the predictive validity of the three MSN models in relation to general intelligence, with previous research showing that the organization of the MSN network (modeled similarly to the MSN_10-feat._) predicted ∼40% variance in WASI (Wechsler Abbreviated Scale of Intelligence) IQ (Seidlitz et al., 2018). The current study did not find a relationship with either a measure of general cognitive functioning or a previously untested domain, executive functioning. Using ninefold cross-validation, no model (at any density) recommended retention of any PLS components. An important strength of the current study is our quantitative approach to cross-validation to confirm the retained number of components; previous studies retained either the single or two components that explained the greatest amount of variance (Seidlitz et al., 2019; Seidlitz et al., 2018). This may mean that previous findings are less generalizable to new datasets, explaining our inability to replicate findings of Seidlitz et al. (2018).

A number of other factors may explain our results. Developmental differences between our sample (healthy young adult population between the third and fourth decades of life) and that of Seidlitz et al. (2018) may account for the lack of predictive ability in our work. Adolescence is a peak period for brain maturation (Gogtay et al., 2004; Sowell et al., 2004), including establishment of cognitive skills such as executive functions, as studied here. This is reflected in data from the NIH Toolbox, in which the total cognition composite highlights shows a greater magnitude of age effects in childhood compared with adulthood (Akshoomoff et al., 2013; Heaton et al., 2014). Throughout childhood, the regions subsuming these functions are reaching structural maturity. Therefore, it is reasonable to believe that it is within the child/adolescent period where the most variance in these neurocognitive skills can be explained by structural networks (as seen by the ∼40% variance in IQ explained by the MSN in Seidlitz et al., 2018).

In the age range that the current study has sampled, the brain should have reached structural maturity (with only subtle age-related effects) and so there is likely less between-individual variance in the MSN. Greater congruence between individual MSNs and the group average MSN in the current study compared with previous adolescent MSNs (correlation of all edge weights: mean *r* = 0.60; Seidlitz et al., 2018) supports this contention. Therefore, the limited variance in the MSN within the age group we studied may mean that there is not enough variance to relate to cognitive functioning.

While we were not able to replicate previous brain-behavior relationships with the MSN, given the evidence above, there is an open hypothesis as to whether the MSN is a valuable tool in independent populations. We therefore propose that the MSN may in fact be a useful phenotype for assessing neuropsychological functioning, but only in populations where there is sufficient variation in the structure of the brain. This may be populations in the infant/child/adolescent period where structural networks are likely to see greatest variability due to developmentally mediated change (such as Galdi et al., 2018; and Seidlitz et al., 2018) or clinical populations where atypical brain structure is seen in the pathophysiology of the disorder (such as Seidlitz et al., 2019; Morgan et al., 2018; and Zheng et al., 2019). It may be the case that these networks hold utility in populations such as these, rather than healthy, matured populations (where measures of brain structure are likely to heavily regress to the mean).

Our analytic approach may also explain the different findings. Seidlitz et al. (2018) used the Wechsler Abbreviated Scale of Intelligence (WASI; Wechsler, 1999), whereas we used the NIH Toolbox Cognition Composite scores (Heaton et al., 2014). The composite score shows high convergent validity with other Wechsler assessments of general intelligence (with the Wechsler Adult Intelligence Scale [WAIS-IV; Wechsler, 2008] *r* = 0.89; Heaton et al., 2014; and with the Wechsler Intelligence Scale for Children [WISC-IV; Wechsler, 2003] *r* = 0.88; Akshoomoff et al., 2013), and therefore core data elements will enable future studies to clarify whether the application of MSNs is relevant to specific measures. We also calculated the MSN at a much lower spatial scale (68 ROIs) compared with previous work (308 ROIs). This lower spatial resolution may result in more regionally specific effects being difficult to detect; however, it may also have allowed us to detect more subtle effects because of increased power. Yet it is important to note that the 308 ROIs are derived by subdividing the 68 ROI atlas used in the current study into equally sized patches and thus still respect the anatomy of the brain in the same way. Therefore, it is highly unlikely that this would explain why we did not replicate previous findings. Future research should replicate the current findings in independent datasets, across different atlases and at different spatial resolutions.

## CONCLUSION

We have demonstrated that when we generate the MSN based on a reduced/limited number of MR features, we produce correlation matrices that are highly similar to those generated with multimodal imaging. However, the nodal-level topology differed based on the number of features. In contrast to previous research, we found that regardless of the number of features, these networks have limited predictive validity of generalized cognitive ability scores, although this may be specific to the current age range under study. Overall, this study provides tentative evidence that in situations where multimodal imaging is not available or clinically/developmentally inappropriate, T1w-restricted MSN construction may be an appropriate proxy for multimodal MSNs. However, nodal-level topology is likely to be biased based upon the neuroanatomical feature sets utilized to construct these networks, limiting generalizability across studies.

## ACKNOWLEDGMENTS

Data were provided (in part) by the Human Connectome Project, WU-Minn Consortium (principal investigators: David Van Essen and Kamil Ugurbil; 1U54MH091657), funded by the 16 NIH Institutes and Centers that support the NIH Blueprint for Neuroscience Research; and by the McDonnell Center for Systems Neuroscience at Washington University.

## SUPPORTING INFORMATION

Supporting information for this article is available at https://doi.org/10.1162/netn_a_00123.

## AUTHOR CONTRIBUTIONS

Dan King: Conceptualization; Data curation; Formal analysis; Methodology; Writing - Original Draft; Writing - Review & Editing. Amanda Wood: Conceptualization; Investigation; Supervision; Writing - Review & Editing.

## FUNDING INFORMATION

Amanda Wood, European Research Council (http://dx.doi.org/10.13039/501100000781), Award ID: 682734.

## TECHNICAL TERMS

Cortical thickness: Distance between the inner (white matter/gray matter boundary) and outer (gray matter/dura boundary) cortical surfaces at each vertex.
Fractional anisotropy: Scalar measuring the degree of directional restriction of water molecule diffusion.
Mean diffusivity: Average diffusion motion of water molecules independent of directionality.
Gaussian curvature: The product of the two principal curvatures at every vertex of the surface, indicating local curvature.
Mean curvature: The mean of the two principal curvatures at every vertex, indicating local folding.
Curvature index: Integrating across all regions of positive Gaussian curvature and dividing by 4*π*.
Folding index: Integral of the product of the maximum principal curvature and the difference between maximum and minimum curvature.
Executive functions: A collection of top-down control processes that allow an individual to be adaptive to novel situations in their environment.
Folding index: Integral of the product of the maximum principal curvature and the difference between maximum and minimum curvature.
