# Supplementary material for: Clinically feasible brain morphometric similarity network construction approaches with restricted magnetic resonance imaging acquisitions
Source: Netw Neurosci. 2020 Mar 1;4(1):274–91. doi: 10.1162/netn_a_00123 (PMC7069065; doi:10.1162/netn_a_00123)
Supplement: Supplementary file 1 [file netn-04-274-s001.pdf]

King, D., & Wood, A. (2020). Clinically-Feasible Brain Morphometric Similarity Network Construction Approaches with Restricted Magnetic Resonance Imaging Acquisitions. *Network Neuroscience*, *4*(1), 274–291. https://doi.org/10.1162/netn_a_00123

Supplementary Materials

Dimension reduction of Executive functioning task performance

A principal component analysis (PCA, using the ‘prcomp’ function in the base R ‘stats’ package (R Core Team, 2016)) was used to find a common EF component across all three EF measures.

Data reduction using the PCA was done for two main reasons; a) to reduce dimensionality, and the number of multiple predictor models being built and b) to ensure that we were predicting (a latent variable of) executive functioning ability, rather than ability linked to task-specific performance.

The PCA suggested a three-component solution, however only the first component had an eigen-value > 1 (eigenvalue=1.607) and so only this component was retained. This component explained ~54% variance across our measures. All three measures; list-sort, card-sort and flanker, positively loaded onto this component (rotated sums of squares loading = .362, .673 and .646 respectively).
